# Insights into Growing Silica Around Monocrystalline Magnetite Nanorods Leading to Colloids with Improved Magnetic Properties—Obstacles and Solutions

**DOI:** 10.3390/nano16030219

**Published:** 2026-02-06

**Authors:** Nele Johanna Künnecke, Irene Morales, Madeleine Alexandra Schaefer, Sebastian Polarz

**Affiliations:** 1 Institute of Inorganic Chemistry, Leibniz University Hannover, Callinstraße 9, 30167 Hannover, Germany; nele.kuennecke@aca.uni-hannover.de (N.J.K.); irene.morales@aca.uni-hannover.de (I.M.); madeleine.schaefer@aca.uni-hannover.de (M.A.S.); 2 Cluster of Excellence PhoenixD (Photonics, Optics and Engineering—Innovation Across Disciplines), Leibniz University Hannover, 30167 Hannover, Germany

**Keywords:** magnetic colloids, shape-anisotropy, silica-coating, core-shell structures, magnetic assembly

## Abstract

Nanoparticles of ferrimagnetic magnetite (Fe_3_O_4_) are cornerstones of modern nanoscience and technology, primarily due to their superparamagnetic behavior. Beyond traditional applications in magnetorheology and magnetic hyperthermia, these materials are increasingly vital in fields like active matter, where precise surface fine-tuning is crucial. While coating isotropic, quasi-spherical magnetite nanoparticles with silica is a well-established and versatile route towards functionalization, transferring this achievement to nanorod systems remains a significant challenge. Successful coating of these high-aspect-ratio geometries would allow to exploit the direction-dependent properties and increased magnetic anisotropies. However, current literature largely focuses on polycrystalline rods composed of small, clustered subunits, which limits their magnetic potential. This work describes a breakthrough in the homogeneous silica coating and stabilization of monocrystalline magnetite nanorods. We demonstrate that the superior magnetic properties of these “naked” monocrystalline rods induce strong dipole-dipole interactions, which trigger aggregation and typically prevent the isolation of individual and homogeneously coated core-shell nanoparticles. By investigating the specific mechanisms of this aggregation, we established a robust coating procedure that yields the desired isolated particles. Critically, we show that the magnetite nanorods retain their monocrystalline integrity within the silica shell, thereby preserving the enhanced magnetic properties of the original nanocrystals.

## 1. Introduction

Magnetic nanoparticle colloids are among the most interesting and well-researched materials in nanoscience, with a wide range of applications, including catalysis [1], environmental remediation [2], and biomedicine [3]. Among the variety of magnetic nanoparticles available, magnetite (Fe_3_O_4_) and maghemite (*γ*-Fe_2_O_3_) nanoparticles in water have attracted significant attention due to their promising applications in biomedicine [4]. This is due to their low toxicity and biocompatibility, the possibility of externally manipulating and guiding them to specific sites by the application of magnetic fields [5], and their ability to generate heat when subjected to radiofrequency fields, leading to their use in magnetic hyperthermia treatments [6,7]. Additionally, these nanoparticles have the potential to function as active colloids with the aim to mimic the autonomy, behavior and complexity of living matter, while their reduced mass and size increases their sensitivity toward external triggers [8,9,10].

It is important to acknowledge the significant influence of nanoparticle shape on the properties of magnetic nanocrystals. The deviation from spherical shape results in direction-dependent properties and enhanced anisotropies [11,12]. In particular, magnetite nanorods are a promising material for magneto-optical [13] and photothermal [14] applications, as well as being of great interest for their application in active colloids due to their directional properties and interactions, enabling more complex and controllable locomotion.

To ensure the applicability of magnetite nanoparticles across different fields, it is essential to have reliable, stable colloidal systems that prevent uncontrolled aggregation. Agglomeration often results in unwanted changes in properties and stability upon clustering [15]. To achieve an easy-to-handle system, coating the magnetite nanoparticles with silica is a common method [16], also known for further increasing the functionalization possibilities of the particles [4,17]. Especially, the coating of quasi-spherical iron oxide nanoparticles with silica using various procedures has been intensively investigated [16,18,19,20,21].

The most widely used technique for equipping magnetite nanocrystals with a silica coating is the sol-gel method, in which silicon alkoxides serve as starting materials for the condensation of a silica shell. In this procedure, condensation occurs on the surface of the nanoparticles, dispersed in an ammoniacal mixture of ethanol/water [18,19]. An approach based on an inverse microemulsion as nanoreactors for the condensation of a silica shell has proved to be very successful. The method provides good control over the shell thickness and colloidal stability of the particles [16]. In the literature, the dynamics of emulsion-based synthesis, used for nanoparticle preparation and coating, are very well described [22], as well for magnetic nanoparticles [23,24]. While the described methods work extremely well for quasi-spherical and cube-like magnetite nanoparticles [25], less is reported about silica-coated nanorods. Instead of starting from monocrystalline magnetite nanorods, routes in the literature synthesize FeOOH nanorods first, followed by coating and the chemical reduction of the core to magnetite in a last step [26,27]. The reduction process causes a decrease in the magnetite nanorod crystallinity, leading to a polycrystalline core structure. Because one cannot control the size of the particles in the polycrystals, their magnetic properties are not as defined as would be possible for monocrystalline systems. The blocking temperatures are broadly distributed, and the materials show lower saturation magnetizations and lack uniaxial anisotropy [26,27,28]. When we tried to adopt the microemulsion-based method [16] and started from monocrystalline magnetite nanorods, we recognized that the yield of stable SiO_2_-Fe_3_O_4_ colloids was below 5% [10].

Because of the strong potential of monocrystalline-Fe_3_O_4_ nanorods coated with SiO_2_, we wanted to understand the reasons for the low yield and develop a more reliable synthesis procedure. Our results are reported in the current paper. The paper is organized as follows. After the preparation of monocrystalline magnetite nanorods (MagNR) by a literature-known procedure [29,30], we investigated the details of the silica coating procedure and indicate potential pitfalls. From the knowledge of the factors that lead to the undesired aggregation of the nanorods in dispersion, important modifications in the synthesis procedures are concluded. The resulting SiO_2_@MagNR colloids were investigated in detail with an emphasis on whether the monocrystalline nature could be retained, along with the effects and benefits for magnetic properties.

## 2. Materials and Methods

**Materials.** All chemicals were used without further purification. They included ammonium hydroxide solution (28–30%, HN_3_-basis, Sigma Aldrich (St. Louis, MI, USA)), cyclohexane (99.5% anhydrous, Sigma-Aldrich (St. Louis, MI, USA)), ethanol (>99.8%, Roth (Karlsruhe, Germany)), hexadecyl amine (Sigma-Aldrich (St. Louis, MI, USA)), Igepal CO-520 (Sigma-Aldrich (St. Louis, MI, USA), M_n_ 441), iron pentacarbonyl (Sigma-Aldrich (St. Louis, MI, USA), >99.99%), methanol (99.9% p.a., Roth (Karlsruhe, Germany)), 1-octanol (99%, Thermo Scientific (Waltham, MA, USA)), oleic acid (90%, Sigma-Aldrich (St. Louis, MI, USA)), sodium chloride (>99.5%, p.a., Roth (Karlsruhe, Germany)), and tetraethoxysilane (TEOS) (98%, Sigma-Aldrich (St. Louis, MI, USA)).

**Synthesis of MagNR.** Magnetite nanorods (MagNR) were synthesized using solvothermal synthesis, as reported by Sun et al. [29]. A mixture of 0.8 g hexadecyl amine, 2.0 mL oleic acid, and 8.0 mL 1-octanol was stirred at 55 °C for 30 min. After cooling to RT, 2.0 mL iron pentacarbonyl was added and stirred for 10 min, then transferred to a Teflon-lined autoclave and heated at 200 °C for 6 h. The nanoparticles were washed with ethanol via centrifugation for 4 times at 9000 rpm for 20 min and stored in 10 mL cyclohexane.

**Conventional synthesis of MagNR-silica nanorods.** The conventional synthesis procedure for coating magnetite with silica is carried as described by LANZ et al. [10]. In 10 mL cyclohexane, 0.5 g Igepal CO-520 have been added and sonicated for 20 min in an ultrasonic bath, before adding 4 mg dispersed MagNR (0.741 mL from the stock solution, at 5.4 mg/mL) and 0.9 mL ammonium hydroxide (28 wt.%). The mixture was stirred for another 20 min, then 160 µL TEOS was added slowly through a syringe pump (50 µL/h). Overnight condensation was terminated by adding 5 mL methanol. The nanoparticles were washed via centrifugation in ethanol for 4 times at 6000 rpm for 20 min and stored in 10 mL ethanol.

**Preparation of inverse microemulsion.** The composition of the inverse microemulsion, used for the silica shell condensation, is based on the synthesis described by Ding et al. [16] with some adjustments. Instead using only ammonia, an electrolyte-ammonia solution was prepared, containing NH_3_ (28 wt.%) and sodium chloride (10^−3^ mol/L). To 40 mL cyclohexane, 1.6 g Igepal CO-520, 8 mg dispersed MagNR (1.481 mL from the stock solution, at 5.4 mg/mL) and 190 µL ammonia solution were added, sonicated in a rosette cell (Supplementary Information, Figure S1) with an ultrasonic probe (Sonoplus HD3100 with MS83 microtip from Bandelin (Berlin, Germany), 100% power) in an ice bath for 60 min. After sonication and formation of the inverse microemulsion, sedimentation was allowed for 60 min before collecting only 25 mL of supernatant (from the 40 mL total) to not disturb the precipitated particles, which still contained approximately 32% of the initial MagNR that was added to the microemulsion. This calculation is based on the ICP measurements of the amount of iron in the initial synthesis of the uncoated MagNRs and the final amount present in the silica-coated MagNRs. The exact yield can slightly vary depending on the size and aspect ratio of the nanorods, and the amount of ligands on the surface (i.e., their initial stability in solution).

**Synthesis of magnetite–silica nanorods.** After the sonication of the inverse microemulsion, the collected supernatant was again sonicated for another 20 min in an ultrasonic bath. Then, 160 µL TEOS was added through a syringe pump (50 µL/h) to 25 mL of the collected supernatant containing the inverse microemulsion with the MagNR. Overnight condensation was terminated by adding 5 mL methanol. The nanoparticles were washed via centrifugation for 4 times at 6000 rpm for 20 min and stored in 10 mL ethanol.

**Synthesis of magnetite spheres.** Magnetite spheres were synthesized as reported by Brunner et al. [28]. First, the iron(III) oleate precursor was synthesized by dissolving 10.8 g iron(III) chloride hexa-hydrate and 36.5 g sodium oleate in a mixture of 80.0 mL ethanol, 140.0 mL hexane and 60.0 mL MilliQ water. After degassing for 20 min, the reaction was heated and refluxed for four hours. The organic phase was washed three times with MilliQ water, dried over MgSO_4_, and the solvent was evaporated to obtain the iron(III) oleate precursor. For the synthesis of the magnetite spheres a mixture of 4.5 g iron(III) oleate precursor, 0.71 g oleic acid and 25.0 g of trioctylamine were heated up to 60 °C and dried under vacuum for 30 min. Afterward, the reaction mixture was heated to reflux (365 °C) with a constant heating rate of 3.3 K/min and stirred for 30 min before cooling down to room temperature. The obtained nanoparticles were precipitated by adding 50.0 mL ethanol and purified via centrifugation for 10 times, at 9000 rpm for 20 min, with a mixture of toluene/ethanol by increasing the amount of toluene with each washing step from 1:7 to 1:3. The particles were then stored in cyclohexane.

**Silica coating of magnetite spheres.** The conventional synthesis procedure for coating magnetite with silica is carried as described by Lanz et al. [9]. In 125 mL cyclohexane, 5 g Igepal CO-520 have been added and sonicated for 20 min in an ultrasonic bath, before adding 60 mg dispersed MagNR (3 mL from the stock solution, at 20 mg/mL) and 0.9 mL ammonium hydroxide (28 wt.%). The mixture was stirred for another 20 min, then 0.5 mL TEOS was added slowly through a syringe pump (50 µL/h). Overnight condensation was terminated by adding 5 mL methanol. The nanoparticles were washed via centrifugation in ethanol for 4 times at 6000 rpm for 20 min and stored in 10 mL ethanol.

**Characterization.** For sonication, the ultrasonic probe Sonopuls HD3100 with MS83 microtip from Bandelin at 100% power was used. Fourier-transform infrared spectroscopy (FT-IR) spectra were obtained with a *Bruker Tensor 27 FTIR* (Karlsruhe/Rheinstetten, Germany), using OPUS. Scanning electron microscopy (SEM/STEM) images were acquired with a *Regulus 8230* from *Hitachi* (Chiyoda, Tokyo, Japan). The samples were prepared by drying the particle dispersion on silicon wafers under ambient conditions. Transmission electron microscopy (TEM) images were obtained with a *Hitachi HT7800* (Chiyoda, Tokyo, Japan) (100 kV), and images were obtained using Radius as well as a FEI Tecnai G2 F20 TMP using Digital Micrograph (Gatan Inc., Pleasanton, CA, USA) to obtain images. The samples were prepared by drop-casting diluted solutions of the nanoparticles into copper grids, letting the solvent evaporate under ambient conditions. Image J was used for size determination for all electron microscope images. Superconducting quantum interference device (SQUID) measurements have been acquired using a *Quantum Design MPMS3* (Pfungstadt, Germany) magnetometer. Zero field-cooled and field-cooled (ZFC-FC) magnetization curves were measured by applying a magnetic field of 100 Oe from 5 to 300 K. Besides, M-H curves were measured at 5 K and 300 K and a maximum applied field of 5 T. All the measurements were done in VSM mode. For calculating the magnetic saturation, the magnetic mass obtained from ICP measurements was used. ICP-OES data were obtained on a Varian Vista AX using the software ICP Expert II (Version 2.0.5). Dynamic light scattering (DLS) measurements were acquired with a *Malvern Zetasizer ZMV 2000* (Worcestershire, UK), to obtain the hydrodynamic size of the MagNR in dispersions and of the micelle sizes of the inverse microemulsion. Thermogravimetric analysis (TG) measurements were performed using a *Netzsch STA 409 PC LUXX* (Selb, Germany) with a heating rate of 5 K/min from 30 °C to 1000 °C in 100% oxygen atmosphere and are used to determine the quantity of organic ligands on the nanoparticle surface. Powder X-Ray Diffraction (pXRD) was performed in transmission on a Stadi-P (STOE) in Debye–Scherrer geometry equipped with a Ge(111) monochromator (Cu-Kα *λ* = 1.54056 Å) and a MYTHEN 1K Stripdetector (DECTRIS, Dättwil-Baden, Switzerland). Measurements were performed from 15° to 80° in 2*theta* with a step width of 1.5. The software WinXPOW (STOE and Cie GmbH, Darmstadt, Germany) was used for data acquisition. All other analysis and all graphic representation were carried out using Origin 2025 and Inkscape.

## 3. Results and Discussion

### 3.1. Monocrystalline Magnetite Nanorods

Because the synthesis of the magnetite nanorods (MagNR) was reproduced from the literature [29], the discussion will be kept short. However, because these particles represent the starting point, a thorough characterization is necessary and is given in the Supplementary Information. The particles were synthesized by solvothermal synthesis (see Exp. Methods). Transmission electron microscopy reveals the high quality of the particles, as shown in Supplementary Information Figure S2a. The average size of the MagNRs is 50 ± 8 nm in length and a width of 9 ± 2 nm, resulting in an aspect ratio of 5.8 (Supplementary Information, Figure S2b). The presence of ligands (oleic acid and hexadecyl amine) can be verified by IR measurements, in which the aliphatic vibrations of the ligands are particularly prominent (Supplementary Information, Figure S2d). From the thermogravimetric measurements (TG), the amount of ligands on the particle surface was determined. A mass loss of 21.4 wt.% was calculated (Supplementary Information, Figure S2f). We have also investigated the dispersion by dynamic light scattering (DLS; Supplementary Information, Figure S2c) and found species with diameters much larger (500–1000 nm) than the individual MagNRs. The latter result is a first indication of the strong tendency to form aggregates in solution, despite the ligand shell.

### 3.2. Microemulsion-Based Method for Silica Shell Generation

**Figure 1:**
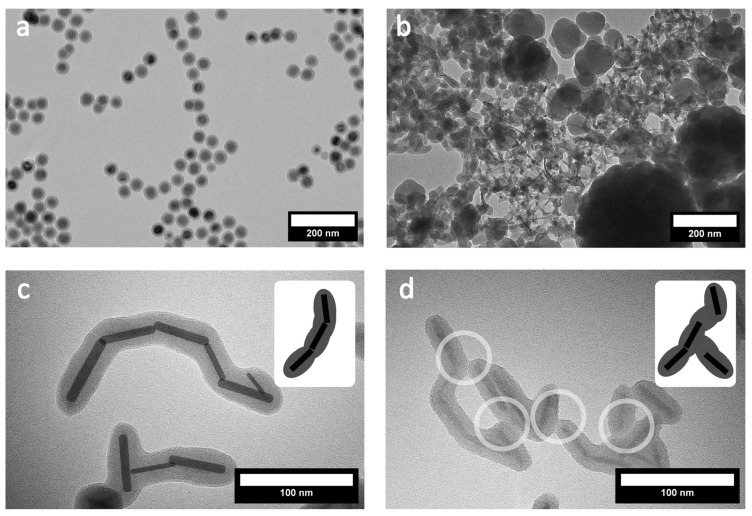
(**a**) TEM image of spherical magnetite nanoparticles after silica coating and (**b**) TEM image of magnetite nanorods after attempted silica coating, both using the conventional inverse microemulsion method. (**c**) Magnetite cores in particle aggregates highlighted in black and (**d**) silica growth connecting particles highlighted by circles, highlighting the most important challenges in the silica coating of MagNR.

As an additional reference state, at first, we prepared silica-coated quasi-spherical particles (MagQS) as described in the literature [9,31,32]. Data are given in the Supplementary Information Figure S3 and Figure 1a. Our results indicate the high reproducibility of the microemulsion-based coating strategy. However, the direct transfer of the methodology to MagNR proved unsuccessful, despite repetitive attempts. Typically, the samples contained mixtures of uncoated MagNR and coated MagNR, together with huge and empty silica particles (Figure 1b). Closer inspection of the silica-coated regions reveals two further, fundamental problems. The MagNRs have a high tendency to attach to each other either by forming direct contacts (Figure 1c), frequently at their tips, or by silica bridges connecting the particles (Figure 1d).

**Figure 2:**
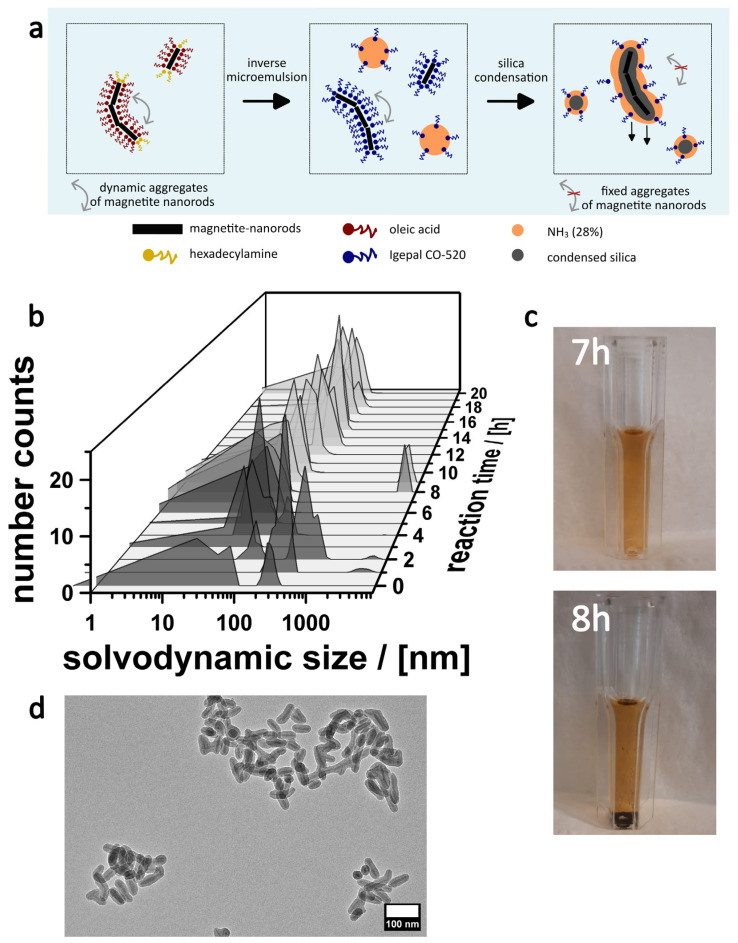
(**a**) Scheme summarizing the analysis of the processes during the microemulsion-based silica coating process of MagNR. (**b**) Particle size distributions derived from the DLS measurements of samples taken at different times. (**c**) Photographic images of dispersion after 7 and 8 h of starting the reaction. (**d**) TEM image of a sample (supernatant) after 20 h.

Because the shape of the magnetite nanoparticles obviously has such an unexpected and significant negative impact, the coating process was investigated in further detail. Dynamic light scattering measurements were taken at different times during the 20 h synthesis (Figure 2). Although one has to be careful with the interpretation of the results, because larger particles dominate the scattering behavior, the measurements indicate that already in the early phases, isolated particles are barely present. Instead, one finds aggregates with sizes less than 100 nm. This observation fits the previously mentioned aggregation process of the pure MagNR dispersion because of their magnetic interactions (Supplementary Information, Figure S2c). Because we have not observed sedimentation of particles during the first 7 h (Figure 2c), we assume that the aggregates are highly dynamic up to this point, and can form and dissolve [33]. A dramatic change is observed after 7 h. One sees that a precipitate has formed, which contains the majority of the MagNRs. The supernatant is still colored, which indicates that there is still a fraction of the particles present with sufficient colloidal stability. According to DLS data (Figure 2b), these stable colloids are in the right range for isolated silica-coated MagNRs. The observation matches the TEM measurements (Figure 1c,d). We conclude that the sol-gel process kicks in after 7 h, fixing the dynamic MagNR aggregates (Figure 2a). The colloidal stability of the aggregates is too low, and they precipitate. Only isolated or sufficiently small aggregates survive the precipitation process, and indeed TEM micrographs of the sample obtained after 20 h indicate nicely coated and less aggregated MagNRs (Figure 2d).

### 3.3. Overcoming the Aggregation of MagNR

**Figure 3:**
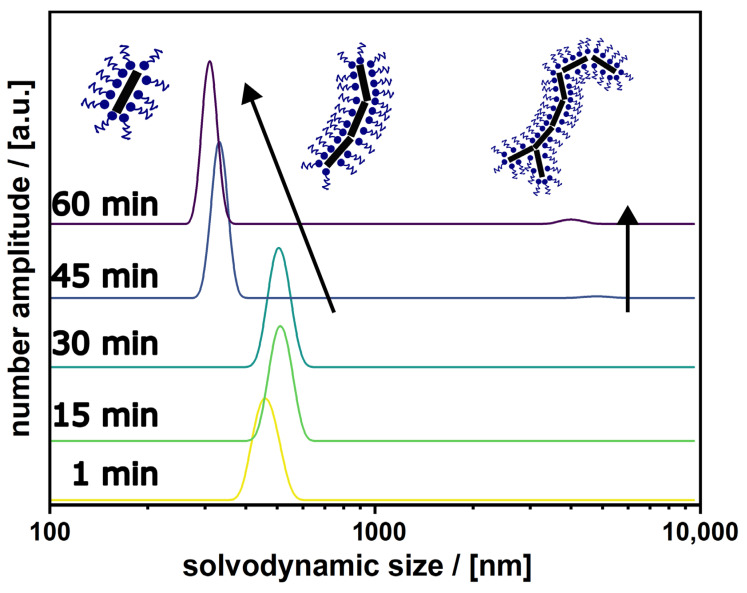
Progress of solvodynamic sizes of MagNR aggregates in the inverse microemulsion over 60 min after sonication is turned off, measured every 15 min. For the first 30 min the most dominant species are midsized aggregates with measured sizes around 500 nm. After 45 min, small aggregates and single particles become more prominent due to sedimentation of the bigger aggregates which are not anymore contributing to the scattering. At the same time a small number of big aggregates with sizes over 3000 nm can be detected during their sedimentation.

The latter observations indicate that it is in principle possible to achieve SiO_2_-MagNRs, but it is essential to increase the colloidal stability of the particles by decreasing the aggregation tendency, even before the beginning of the silica coating process. If our assumption is correct, that the initial aggregates (Figure 2a) are dynamic, a higher degree of particle separation could be achieved by introducing greater kinetic energy in the system prior to the sol-gel process, so during the preparation of the inverse microemulsion (see Materials and Methods) Our idea is that the use of high-power ultrasound as a pre-treatment of the MagNRs in the inverse microemulsion can break up the aggregates. Therefore, we used a sonication probe placed directly in the solution using a special glass vial (rosette vessel as shown in the Supplementary Information, Figure S1). The ultrasound was applied for one hour and turned off, and the change in aggregate sizes after sonication was investigated using DLS. The results are shown in Figure 3. Initially, during the first 30 min after sonication, there are aggregates that grow in size. This growth is presumably caused by the dynamic nature of the aggregation and the nanoparticle magnetic interactions which play an important role, once the energy introduced by the sonication is removed. However, after 45 min one observes significantly smaller aggregates due to the fast precipitation of undispersed aggregates. Only a very small signal for large agglomerates with sizes larger than 1000 nm is present, which can be separated. Overall, for subsequent coating, the remaining supernatant after 60 min is taken, containing the disaggregated and stable nanorods.

**Figure 4:**
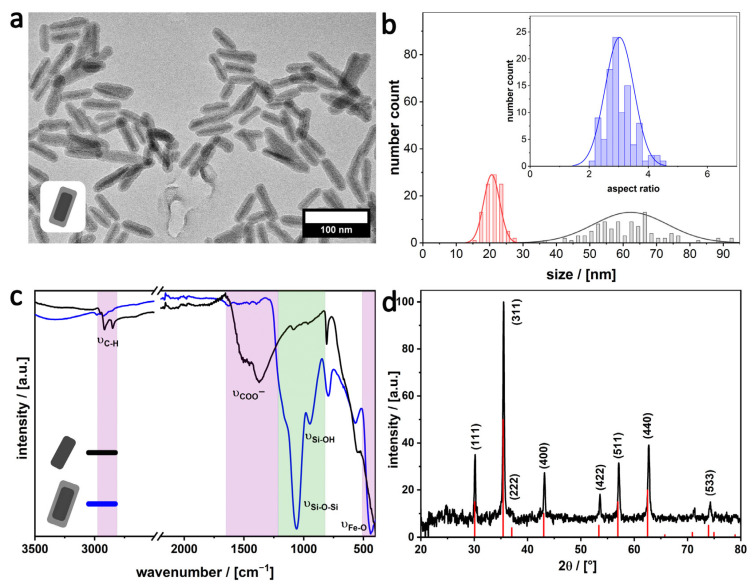
(**a**) TEM images of silica coated MagNR, (**b**) histogram including length of 62 ± 11 nm (grey), width of 21 ± 2 nm (red) and an aspect ratio of 3.0 ± 0.5 (inset, blue), measured from 100 particles in Image J. (**c**) IR-spectra of MagNR (black) and silica coated MagNR (blue). Characteristic vibrations are highlighted for organic ligands (pink) and silica (green). (**d**) PXRD of silica-coated MagNR (black) with indication of literature-found reflexes (red) for magnetite (JCPDS 19-0629).

For avoiding that the particles fuse due to condensation via the growing silica shell (Figure 1d), we refer to papers describing that the addition of electrolytes to a sol-gel process enhances the inter-particle repulsion [34,35]. Combining this with the sonication treatment leads to a profound increase in the quality of the sample seen from TEM images shown in Figure 4a. Defined and isolated SiO_2_-coated MagNRs with a length of 62 ± 11 nm, width of 21 ± 2 nm, and an aspect ratio of 3.0 ± 0.5 are observed (Figure 4b), and scanning electron microscopy data shown in Supplementary Information Figure S4. The silica shell has a thickness of 13 ± 2 nm.

The shell consists of silica, as confirmed by Fourier transform infrared (FT-IR) spectroscopy (Figure 4c). The characteristic Si-O-Si and Si-OH stretching vibrations can be observed at 1060 and 1160 cm^−1^ [36]. Furthermore, one of the Fe-O vibrations of the magnetite can be detected at 548 cm^−1^. The FT-IR data also indicate that the ligands, which were initially bound to the surfaces of MagNR after their synthesis, were cleaved off. Most importantly, the COO^−^ vibration in the 1504 cm^−1^ range associated with the oleate groups and the aliphatic CH valence vibrations (3000–3200 cm^−1^) have vanished [29].

**Figure 5:**
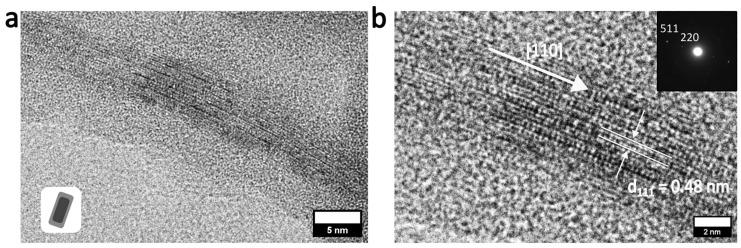
HR-TEM images of (**a**) a single silica coated MagNR and (**b**) markup of lattice planes from magnetite [110] with distance of 0.48 nm, characteristic for magnetite. Inset showing the diffraction reflexes in reciprocal space [30,37].

Analysis of the sample with powder X-ray diffraction (pXRD; Figure 4d) indicates that the core still consists of magnetite. The pattern looks almost identical to the pattern of MagNR (Supplementary Information, Figure S2d). In particular, the reflexes are very narrow, which means that the crystallinity of the nanorods has not been changed due to the sonication procedure. Final proof that the monocrystalline character could be retained comes from high-resolution (HR) TEM and electron diffraction measurements shown in Figure 5. The crystal growth of the monocrystalline MagNR core falls along with the easy magnetic axis of magnetite, following the [110] direction. The measured distance (111) is 0.48 nm (Figure 5b), which corresponds to the literature [30], reported spacing of the interplanar d_111_ distance, standing vertically on the [110] axis, forming the orthogonal cubic lattice system of magnetite.

**Scheme 1:**
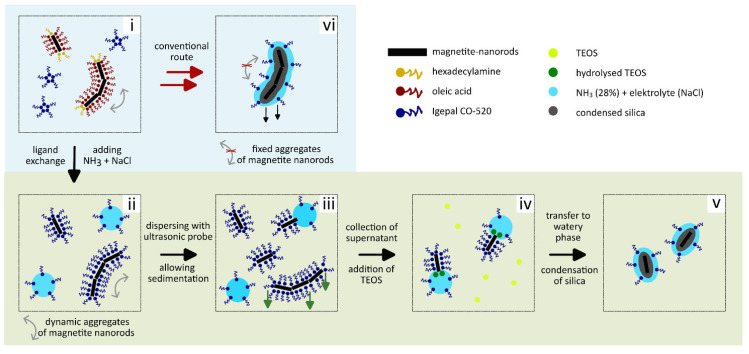
Silica coating mechanism for MagNR. (**i**) Dynamic MagNR aggregates in cyclohexane undergoing (**ii**) a ligand exchange with Igepal CO-520 forming the inverse microemulsion when NH_3_ is added. (**iii**) Due to ultrasonication the aggregates are broken up and (**iv**) remaining aggregates are separated by sedimentation and collection of the supernatant containing the well dispersed individual MagNR, which pass into the inner micelles when TEOS is added. (**v**) Inside the micelles the hydrolyzed TEOS is arranged on the NR surface and then forms a dense silica-shell when condensation onto the NR surface. (**ii**–**iv**) The pre-treatment before adding TEOS prevents (**vi**) fused core shell parts with multiple cores.

Scheme 1 summarizes improvements in the synthesis method and the entire process. The ligands, used for the formation of the inverse microemulsion can exchange the surface ligands of the pristine MagNRs. After the sonication with the ultrasonic probe, most of the aggregates are broken up and kept separate. In the same step, electrolytes are included to prevent the silica shells from growing together during silica condensation. After being added, the silica precursor will hydrolyze and condense on the MagNR surface to form a uniform silica layer. The ability to preserve the crystalline properties of the magnetite cores even during the silica coating process demonstrates a major advantage of the presented synthesis method over other approaches. Additionally, the silica shell evenly coats the magnetite rods making this core shell particle an attractive base for various anisotropic particle systems.

**Figure 6:**
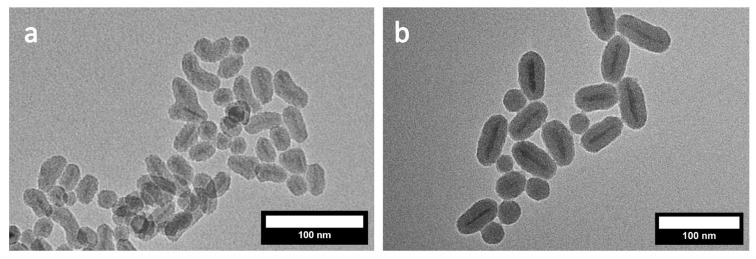
TEM images of silica-coated MagNR with lengths of (**a**) 24 nm and (**b**) 43 nm.

The reproducibility of the coating procedure to obtain distinct and homogeneously single-core-coated MagNRs becomes apparent when the 50 nm MagNR were again coated with silica. In that case, a uniform silica coating with a thickness of 16 nm (Supplementary Information, Figure S5a,b) could be achieved, which demonstrates the reliability of the presented route. To investigate the versatility of the coating procedure, smaller MagNR with lengths (aspect ratio) of 43 ± 9 nm (5.7) and 24 ± 6 nm (4.2) were synthesized (Supplementary Information, Figure S6). The application of the reported synthesis method (Scheme 1) delivers silica coated MagNRs also for these samples as shown in Figure 6.

### 3.4. Magnetic Properties of Silica-Coated MagNR

**Figure 7:**
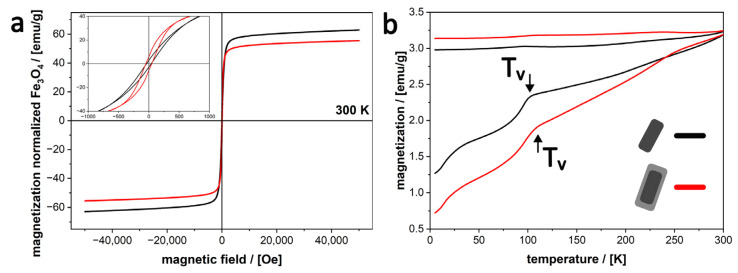
(**a**) Magnetization curves of MagNR (black) compared to silica coated MagNR (red) at 300 K normalized to magnetite mass. (**b**) ZFC-FC curves, with Verwey transition at 94 K for MagNR and 97 K for MagNR@SiO_2_ (from first derivative).

To investigate the influence of the silica shell on the magnetic properties, hysteresis cycles (MH curves) and ZFC-FC (M vs. T) measurements of both the uncoated MagNR (51 nm) and resulting silica-coated MagNR were conducted. The magnetic properties (Figure 7) of the MagNR are only minimally impacted by the shell addition. Interestingly, the saturation magnetization of our silica-coated nanorods is more than three times higher (20 emu/g, Supplementary Information, Figure S7a) than that of similarly coated spherical magnetite nanoparticles previously described in literature [9]. This enhancement is observed despite the spherical cores having a magnetic volume approximately 3.5 times larger, indicating the high monocrystallinity of our nanorods and a significant reduction in the “magnetic dead layer” and crystalline defects, properties that are preserved even after the silica coating process.

The superior performance of nanorod-shaped nanoparticles in comparison to their spherical counterparts is further evidenced by the comparison of the remanence M_R_. At 5 K, our coated MagNR exhibited an M_R_ of approximately 5 emu/g, a five-fold increase over the 1 emu/g reported for the abovementioned silica-coated magnetite spheres [9]. While the relative mass of the silica shell influences the total magnetization, such a pronounced difference in remanence is primarily a consequence of the increased shape anisotropy of the nanorod system. Unlike isotropic spheres, which exhibit no preferred magnetization axis, these nanorods possess a “hard” and “easy” axis, allowing for significantly better performance in applications requiring oriented magnetic response, providing as well site-selective reactivity arising from the anisotropic distribution of crystal facets exposed to the solvent [38].

When investigating the effect of the silica coating on the MagNR, both, the coated and uncoated MagNR, display an almost superparamagnetic behavior, with very low coercive fields (H_c_) at room temperature (Supplementary Information, Figure S7a). While the overall saturation magnetization (M_S_) at RT is decreased by 54.4% due to the diamagnetic silica shell, the relative M_S_ (normalized to magnetite mass) only decreases by 11.8% (Figure 7a). This is consistent with an increased surface anisotropy and surface spin disorder resulting from the silica condensation onto the MagNRs [39]. The almost superparamagnetic behavior of the silica coated MagNR, could also be recognized for the reproduced synthesis, when compared to the initial synthesis (Supplementary Information, Figure S8a).

Furthermore, the Verwey transition (T_v_) was observed at 94 K and 97 K in the as-synthesized and coated MagNRs, respectively, detected as a kink in the ZFC-FC curve (Figure 7b). The Verwey transition is exclusive to magnetite and is highly sensitive to stoichiometry, disappearing entirely upon oxidation into maghemite [40]. Its clear presence confirms that the MagNRs consist primarily of magnetite, both before and after the silica-coating procedure. This indicates that the microemulsion process does not induce significant oxidation, preserving the magnetic phase of the MagNRs. While the T_V_ for bulk magnetite is typically 120 K [41], the shift to lower temperatures observed here is consistent with finite-size effects and the reduction of the magnetic core to the nanoscale [41]. The slightly higher T_v_ observed in the case of the silica coated MagNRs, indicates that the silica shell acts as a protection layer reducing its oxidation in comparison to the as-synthesized MagNRs. However, the ZFC-FC curve (Figure 7b) shows that the blocking temperature is not yet reached at 300 K, so the MagNRs (as-synthesized and silica-coated) are in the ferrimagnetic-superparamagnetic limit within this temperature range.

We want to highlight the distinct advantages of the monocrystalline cores structure in terms of its magnetic properties, in comparison to the polycrystalline systems that are frequently mentioned in literature [26,27,42]. Monocrystallinity maximizes effective magnetic anisotropy by aligning the intrinsic magnetocrystalline easy axis uniformly with the dominant shape anisotropy. Furthermore, the absence of grain boundaries eliminates critical magnetic defects and pinning sites, resulting in higher saturation magnetization (M_s_) for a given magnetic volume and, crucially, a more predictable coercive field (H_c_). By controlling the nanorod volume, the resulting system exhibits near-superparamagnetic behavior at room temperature, making it the ideal option for reversible manipulation in biological environments, magnetic fluids and active colloids. This state ensures that magnetic manipulation can be stopped immediately when the field is turned off due to negligible remanence, effectively preventing agglomeration and residual movement while still generating a strong response to applied fields.

Overall, it can be concluded that it is possible to preserve the magnetic properties of the particles after silica coating, with the advantage of the protection offered by the silica towards external factors leading to oxidation of the material. In correlation with their small size, the core shell particles with monocrystalline MagNR cores could be magnetically manipulated, and later applications can benefit from their direction-dependent magnetic properties.

## 4. Conclusions

In this work, we described a coating process which allows for obtaining individual silica-coated MagNR particles with a single monocrystalline MagNR as a core. Among the benefits of this method are the reproducibility and versatility, the uniformity of the coating, and the preservation of the monocrystalline and magnetic properties of the MagNRs. The key step to obtain such individual particles from the silica coating process is to perform an ultrasonic treatment prior to the condensation of the silica. Additionally, electrolytes were introduced to obtain better separation between the silica shells. It was observed in DLS measurements that the MagNR tend to form aggregates which will then be fixed to big, tip-to-tip connected particles during the condensation of the silica shell if no other action is taken. To overcome this challenge, the magnetite rods together with the inverse microemulsion have to be sonicated with an ultrasonic probe in order to achieve optimal dispersion of the nanorods, followed by a precipitation step. This allows for the collection of well dispersed magnetite nanorods, to uniformly growth a silica shell around. The presented method can be applied on magnetite NR of various lengths, enabling the functionalization of nanorods with varying degrees of anisotropy. The resulting system preserves the monocrystallinity of the magnetite rods, thereby maintaining their magnetic properties, and thus emphasizes the benefits of the particles obtained by this method in comparison to the polycrystalline systems known in the literature. This opens up numerous possibilities for applications, such as designing anisotropic active colloids that benefit from the directional properties of MagNR core and enable multiple further functionalization possibilities.

## Data Availability

The authors state that the data supporting the results of this study can be found within the paper and the ESI. If raw data are required, they can be provided by the corresponding author upon reasonable request.

## Supplementary Materials

The following supporting information can be downloaded at: https://www.mdpi.com/article/10.3390/nano16030219/s1. In the supplementary information complementary TEM and SEM images, IR spectra, PXRD, DLS, TGA, and SQUID measurements can be found.
