# Supplementary material for: Insights into Growing Silica Around Monocrystalline Magnetite Nanorods Leading to Colloids with Improved Magnetic Properties—Obstacles and Solutions
Source: Nanomaterials (Basel). 2026 Feb 6;16(3):219. doi: 10.3390/nano16030219 (PMC12899821; doi:10.3390/nano16030219)
Supplement: Supplementary file 1 [file nanomaterials-16-00219-s001.zip › nanomaterials-4081946-supplementary.pdf]

nanomaterials

Supplementary Material

Insights into Growing Silica Around Monocrystalline Magnetite Nanorods Leading to Colloids with Improved Magnetic Properties—Obstacles and Solutions

Nele Johanna Künnecke [1], Irene Morales [1,2], Madeleine Alexandra Schaefer [1] and Sebastian Polarz [1,*]

[1] Institute of Inorganic Chemistry, Leibniz University Hannover, Callinstraße 9, 30167 Hannover, Germany; nele.kuennecke@aca.uni-hannover.de (N.J.K.); irene.morales@aca.uni-hannover.de (I.M.); madeleine.schaefer@aca.uni-hannover.de (M.A.S.)
[2] Cluster of Excellence PhoenixD (Photonics, Optics and Engineering - Innovation Across Disciplines), Leibniz University Hannover, 30167 Hannover, Germany
* Correspondence: sebastian.polarz@aca.uni-hannover.de; Tel.: +49 11 762 2254

Academic Editor(s): Fabien Grasset

Received: 19 December 2025
Revised: 3 February 2026
Accepted: 3 February 2026
Published: 6 February 2026

Copyright: © 2026 by the authors. Submitted for possible open access publication under the terms and conditions of the Creative Commons Attribution (CC BY) license.

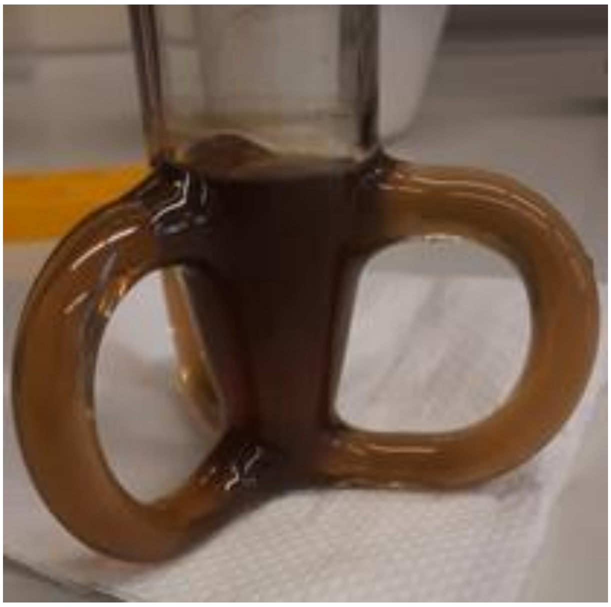

Figure S1. Rosette cell, used for ultrasonic treatment of particle dispersions with an ultrasonic probe.

https://doi.org/10.3390/nano16030219

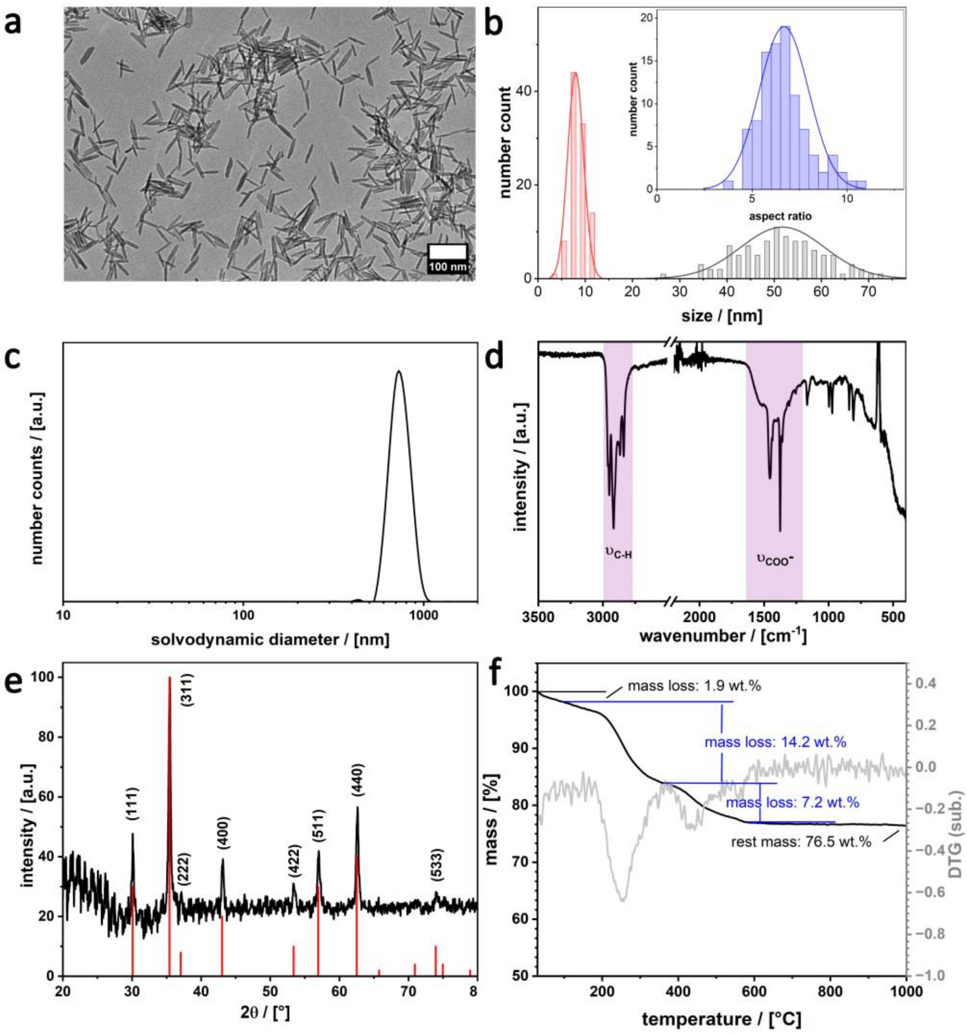

Figure S2. (**a**) TEM image of the magnetite NR, used in core-shell particle. (**b**) Histogram of particle with 50 ± 8 nm length (grey), width of 9 ± 2 nm (red) and an aspect ratio of 5.8 (inset, blue), measured from 100 particles in Image J. (**c**) Solvodynamic diameter of MagNR (5 mg/mL) in cyclohexane from DLS measurements repeated five times, with an average size of the aggregates between 620 and 750 nm. (**d**) IR spectrum of MagNR and the surface ligands oleic acid and hexadecyl amine used in the synthesis. Prominent CH vibrations from ligands marked in pink. (**e**) PXRD of MagNR (black) with indication of literature found reflexes (red) for magnetite (JCPDS 19-0629). (**f**) Amount of ligands on MagNR surface, mass loss of overall 21.6 wt.% organic ligands oleic acid and hexadecyl amine(blue) in two steps of 14.2 wt.% at 90-360 ° C (absorbed ligands) and 7.2 wt.% at 360-570 °C (bond ligand), measured in TG-analysis.[29]

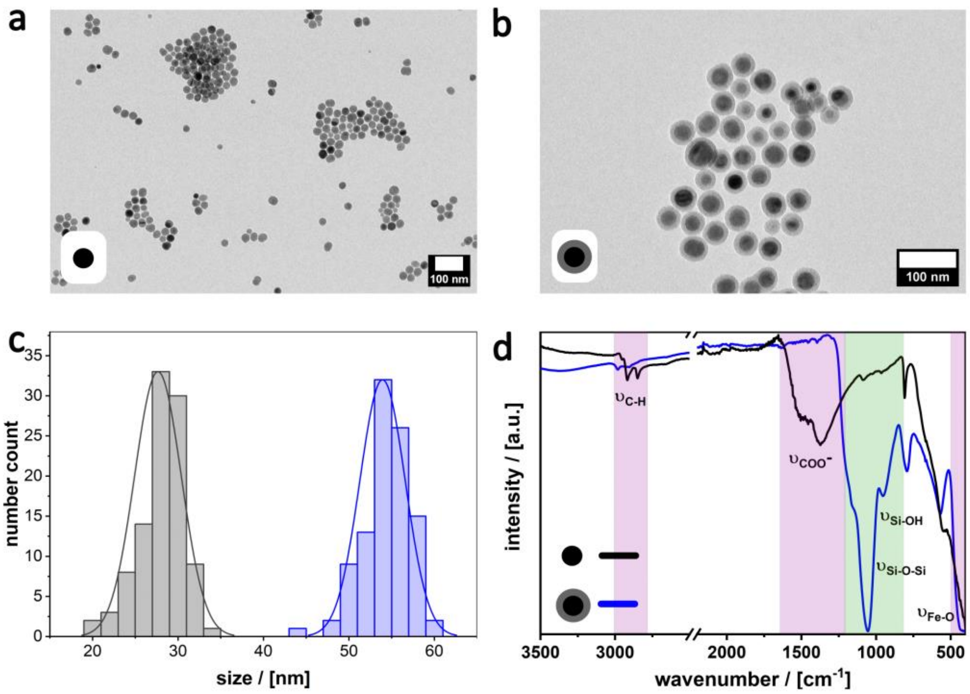

Figure S3. TEM image of (**a**) magnetite spheres with a diameter of 28 ± 3 nm and (**b**) silica coated magnetite spheres with a diameter of 54 ± 3 nm. The particles have a silica shell thickness of 13 ± 3 nm and were synthesized following the procedures described below. (**c**) Histogram of particle diameters of magnetite spheres (grey) and silica coated magnetite spheres (blue), measured from 100 particles in Image J. (**d**) IR spectrum of MagNR and the surface ligands oleic acid and hexadecyl amine used in the synthesis. Prominent CH vibrations from ligands marked in pink.

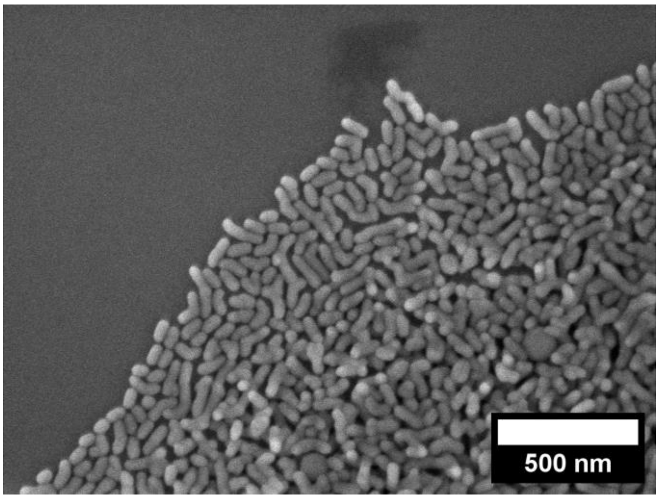

Figure S4. SEM images of silica coated MagNR, using the advanced synthesis method including sonication, precipitation and electrostatic stabilization.

https://doi.org/10.3390/nano16030219

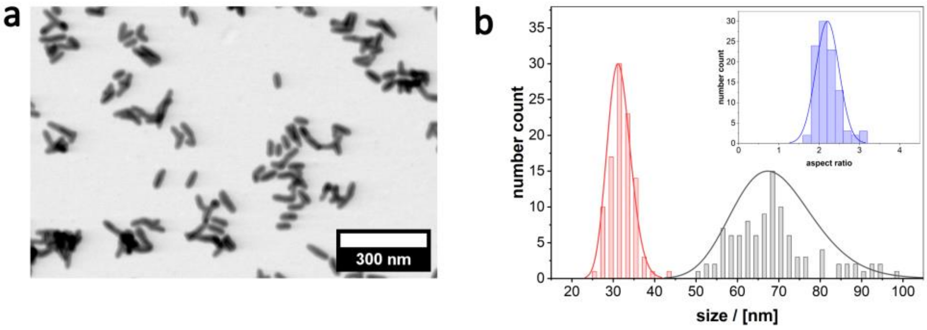

Figure S5. (**a**) STEM image of silica coated MagNR from reproductive shell synthesis. (**b**) Histogram of silica coated MagNR, from reproductive shell synthesis, with 69 ± 10 nm length (grey), width of 32 ± 3 nm (red) and an aspect ratio of 2.2 (inset, blue), measured from 100 particles in Image J.

https://doi.org/10.3390/nano16030219

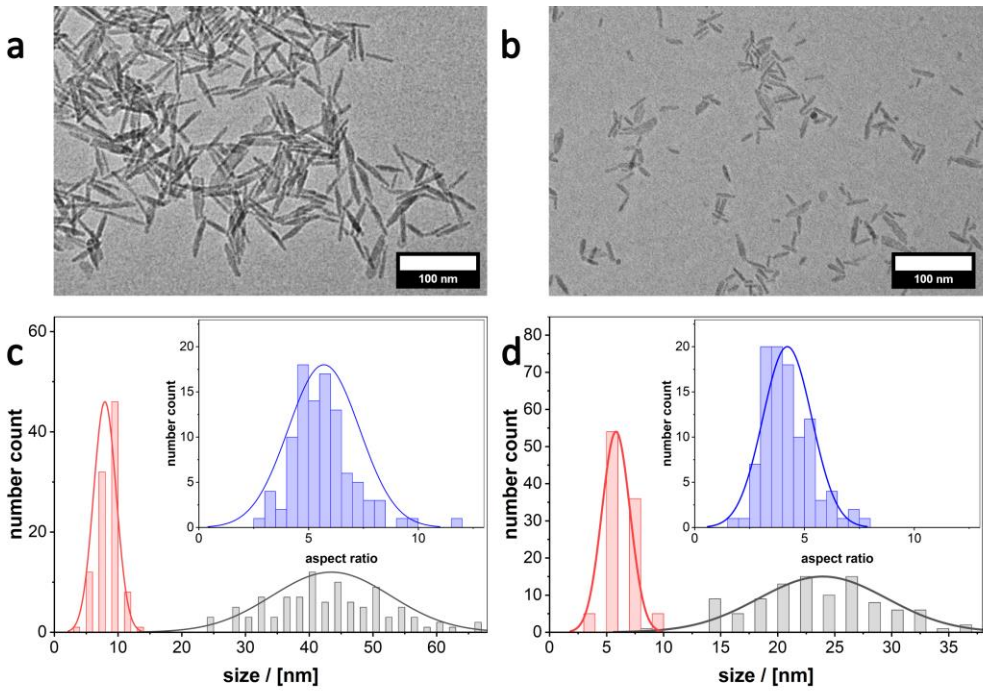

Figure S6. TEM images of (**a**) MagNR with a length of 43 ± 9 nm, width of 8 ± 2 nm and an aspect ratio of 5.7 ± 1.6. **(b**) MagNR with a length of 24 ± 6 nm, width of 6 ± 1 nm and an aspect ratio of 4.2 ± 1.1 (scalebar = 100 nm). Histograms of particle lengths for (**c**) 44 nm particles and (**d**) 25 nm particles, measured from 100 particles in Image J. Synthesis of different lengths due to variation of ligand ratio.[29]

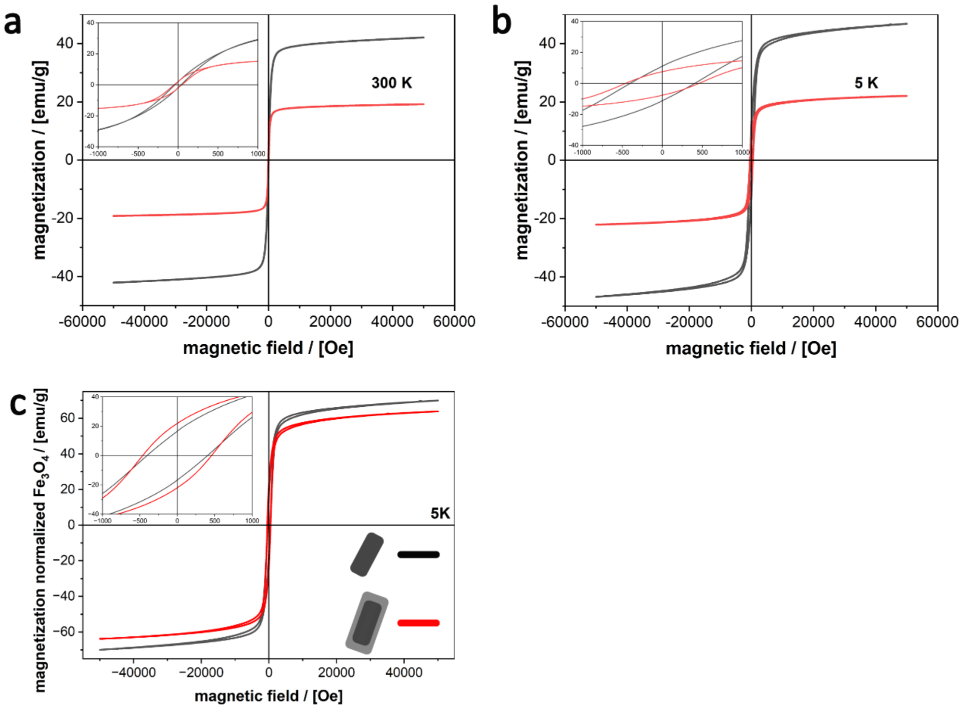

Figure S7. Magnetization curves as measured of MagNR (black) and silica coated MagNR (red) at (**a**) 300 K and (**b**) 5 K. Total mass consisting of MagNR mass and either mass of ligands on surface on MagNR or the mass of the silica shell. Magnetization curves at (**c**) 5K normalized to magnetite mass.

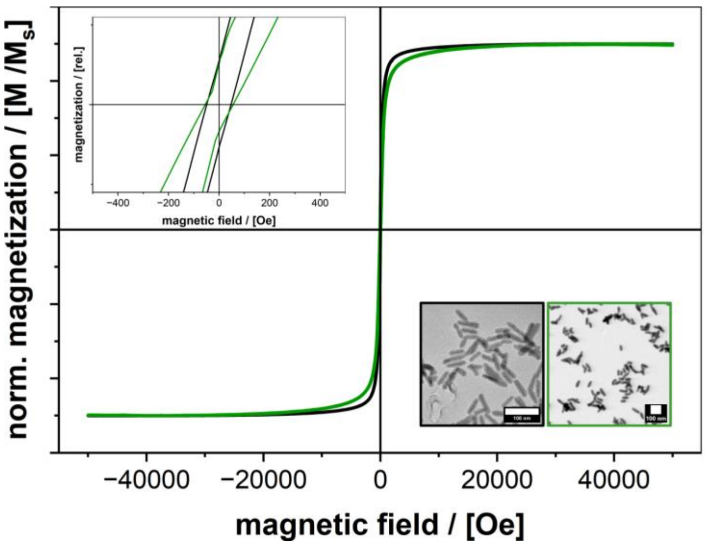

Figure S8. Magnetization curves of initial silica coating synthesis (black) and reproduction of silica coating synthesis (green) at 300 K.

https://doi.org/10.3390/nano16030219

References

29. Sun, H.; Chen, B.; Jiao, X.; Jiang, Z.; Qin, Z.; Chen, D. Solvothermal Synthesis of Tunable Electroactive Magnetite Nanorods by Controlling the Side Reaction. *J. Phys. Chem. C* **2012**, *116*, 5476–5481, doi:10.1021/jp211986a.
